# Quality of life in patients who underwent ^125^I brachytherapy, ^125^I brachytherapy combined with three-dimensional conformal radiation therapy, or intensity-modulated radiation therapy, for prostate cancer

**DOI:** 10.1093/jrr/rry101

**Published:** 2018-12-20

**Authors:** Yasushi Nakai, Nobumichi Tanaka, Isao Asakawa, Satoshi Anai, Makito Miyake, Shunta Hori, Yosuke Morizawa, Yoshihiro Tatsumi, Masatoshi Hasegawa, Tomomi Fujii, Kiyohide Fujimoto

**Affiliations:** 1 Department of Urology, Nara Medical University, 840 Shijo-cho, Kashihara-shi, Nara, Japan; 2 Department of Radiation Oncology, Nara Medical University, 840 Shijo-cho, Kashihara-shi, Nara, Japan; 3 Department of Diagnostic Pathology, Nara Medical University, 840 Shijo-cho, Kashihara-shi, Nara, Japan

**Keywords:** brachytherapy, brachytherapy with three-dimensional conformal radiation therapy, prostate cancer, intensity-modulated radiation therapy, Quality of Life

## Abstract

The purpose of this study was to evaluate quality of life (QOL) in prostate cancer patients treated with ^125^I brachytherapy (BT), ^125^I brachytherapy combined with 3D conformal radiation therapy (BT+3D-CRT), or intensity-modulated radiation therapy (IMRT). We evaluated disease-related QOL in patients who underwent BT, BT+3D-CRT, or IMRT, using the Expanded Prostate Cancer Index Composite questionnaire before treatment and at 3 and 24 months post-treatment. Multivariate analyses were conducted to determine factors associated with a minimum important difference (MID) in urinary, bowel, sexual, and hormone domain scores at 3 and 24 months post-treatment. Of 558 enrolled patients (IMRT, 123; BT, 230; and BT+3D-CRT, 205), urinary domain scores showed a MID after BT, BT+3D-CRT and IMRT at 3 months in 69%, 84% and 25% of patients, respectively, and at 24 months in 43%, 54% and 28% of patients, respectively. On multivariate analysis, BT+3D-CRT [3 months: odds ratio (OR) = 12.7; *P* < 0.001; 24 months: OR = 3.29; *P* = 0.001] and BT (3 months: OR = 6.28; *P* < 0.001 and 24 months: OR = 2.22; *P* = 0.027) were associated with more severely worsened urinary QOL than IMRT. Bowel domain scores showed a MID at 3 months after BT, BT+3D-CRT, and IMRT in 37%, 68% and 41% of patients, respectively, and at 24 months in 29%, 46% and 43% of patients, respectively. On multivariate analysis, BT+3D-CRT (3 months: OR = 4.20; *P* < 0.001 and 24 months: OR = 2.63; *P* < 0.001) and IMRT (24 months: OR = 1.98; *P* = 0.029) were associated with more severely worsened bowel QOL than was BT. Information about the changes in QOL outcomes associated with radiotherapy modalities could guide treatment decisions.

## INTRODUCTION

Currently, we can use several definitive treatment modalities such as ^125^I brachytherapy (BT), BT combined with external-beam radiation therapy (EBRT), and intensity-modulated radiation therapy (IMRT) for localized and locally advanced prostate cancer. Each modality has advantages and disadvantages, and exerts a distinct impact on quality of life (QOL), particularly with regard to urinary, bowel, sexual and hormonal health [1–5]. Evans *et al.* found that BT worsened urinary-related QOL, compared with IMRT and stereotactic body radiotherapy (SBRT) [3]. However, the study did not include a BT and EBRT combination therapy group (this option is beneficial in intermediate-risk and high-risk prostate cancer). Amini *et al.* reported that compared with EBRT alone, combination therapy with BT and EBRT decreased the risk of death in intermediate- and high-risk prostate cancer (75.6–81 Gy) [5]. Recently, the Androgen Suppression Combined with Elective Nodal and Dose Escalated Radiation Therapy (ASCENDE-RT) trial showed a progression-free survival benefit of the combination therapy with BT and EBRT (hazard ratio = 0.473; *P* = 0.0022) [6]. These results showed the superiority of combination therapy with BT and EBRT in patients with intermediate-risk and high-risk prostate cancer.

Due to the impact of urinary, rectal, sexual and hormonal side effects of radiation treatment [1–5], QOL changes after each of the radiotherapy options are important to consider. The Expanded Prostate Cancer Index Composite (EPIC) is a validated tool that measures disease-related QOL in four domains relevant to patients with localized prostate cancer [7]. Morton *et al.* reported that the EPIC was a more sensitive tool for detecting effects on function and bother than were the generic toxicity scales [8, 9]. However, to our knowledge, there are no reports comparing QOL in groups receiving IMRT, BT, or the combination therapy with BT and EBRT. Thus, we aimed to evaluate and compare QOL after BT, after BT combined with 3D conformal radiation therapy (BT + 3D-CRT), and after IMRT, using the EPIC questionnaire.

## MATERIALS AND METHODS

### Patients

This study was conducted in accordance with the provisions of the Declaration of Helsinki (59th World Medical Association General Assembly, Seoul, Korea, in October 2008), and the study protocol was approved by the ethics committee. Data on disease-related QOL in patients who underwent BT, BT + 3D-CRT, and IMRT from April 2010 to March 2014 at the Nara Medical University was collected (prospectively). All patients who received radiation therapy during the study period were offered the opportunity to participate in this study. The study aims and methodologies were explained, and the questionnaire and a leaflet about this study were given to each patient. All patients who answered the questionnaire were enrolled in this study.

### Radiation therapy

The BT group was treated by seed implantation alone at a dose of 160 Gy, whereas the BT + 3D-CRT group was treated at a dose of 110 Gy. The target portion of 3D-CRT was determined 1 month after seed implantation, and patients received a cumulative dose of 45 Gy divided into 25 fractions (1.8 Gy per fraction) using 10 MV photon beams. The clinical target volume included the entire prostate and the proximal third of the seminal vesicles [10]. IMRT was given at a dose of 74–76 Gy in 2-Gy fractions with a 1-cm or 6-mm margin at the prostate–rectum interface. In general, elective lymph node irradiation was not routinely employed during this study period, and patients did not undergo pretreatment fiducial marker placement for image guidance during daily fractions.

### Quality of life

EPIC questionnaires were given to enrolled patients before treatment (i.e. baseline) and at 1, 3, 6, 12 and 24 months post-treatment. A minimally important difference (MID), or a lack of return to baseline, was defined as a parameter value that was greater than half a standard deviation from its baseline value, in all patients who underwent the particular treatment [1, 11].

### Variables

The prostate-specific antigen (PSA) value at diagnosis and the prostate volume measured during prostate biopsy were used for analysis. Uroflowmetry was performed within the month preceding each radiation treatment, and the maximum flow rate (Qmax) and the post-void residual (PVR) were used as parameters in this study.

### Statistical analysis

Statistical analysis was performed using SPSS for Windows (version 20.0; IBM, Armonk, NY, USA). The Mann–Whitney *U*-test was used for continuous variables, and the chi-square test was used for categorical variables. Multivariate logistic regression analysis was used to identify predictive factors for the occurrence of lowering the scores by a MID or more from baseline scores at 3 and 24 months. Among the BT, BT + 3D-CRT, and IMRT groups, the group with the lowest percentage of patients displaying a MID was chosen as the reference. A *P* value of < 0.05 was considered statistically significant, and the Bonferroni correction for multiple comparisons was used where appropriate.

## RESULTS

### Characteristics of enrolled patients

The numbers of patients enrolled were 141 for IMRT, 235 for BT, and 213 for BT + 3D-CRT. At baseline, the EPIC questionnaire was completed for 123 patients treated with IMRT, for 230 patients treated with BT, and for 205 patients treated with BT + 3D-CRT. The mean age of the IMRT group was significantly older than that of the BT (*P* < 0.001) and the BT + 3D-CRT (*P* < 0.001) groups; the mean age of the BT + 3D-CRT group (*P* < 0.001) was significantly older than that of the BT group. At diagnosis, compared with the BT group, the BT + 3D-CRT (*P* < 0.001) and the IMRT (*P* < 0.001) groups had higher PSA values; the IMRT group (*P* < 0.001) had a higher PSA value than the BT + 3D-CRT group at diagnosis. The prostate volume of the BT + 3D-CRT group was significantly smaller (*P* < 0.001) than that of the BT group, whereas the prostate volume of the IMRT group was smaller than that of the BT group (*P* = 0.002). The proportion of high-risk patients, by National Comprehensive Cancer Network (NCCN) classification criteria in the IMRT, BT, and BT + 3D-CRT groups was 47.1%, 1.7% and 42.9%, respectively. The proportion of intermediate-risk patients by NCCN classification criteria in the IMRT, BT, and BT + 3D-CRT groups was 43.0%, 52.1% and 55.1%, respectively. The proportion of low-risk patients by NCCN classification criteria in the IMRT, BT, and BT + 3D-CRT groups was 9.8%, 46.1% and 2.0%, respectively. The mean maximum flow rate of the IMRT group was significantly lower than that of the BT (*P* < 0.001) and the BT+3D-CRT (*P* < 0.001) groups. The mean PVR value was significantly higher in the IMRT group than in the BT (*P* < 0.001) and the BT+3D-CRT (*P* < 0.001) groups (Table 1).

**Table 1.** Baseline characteristics of patients with prostate cancer who underwent radiation therapy

	IMRT	BT	BT + 3D-CRT
Median (range) or *n*	(*n* = 123)	(*n* = 230)	(*n* = 205)
Age, years	73 (52–82)	69 (48–81)**	70 (49–84)**^††^
PSA, ng/ml	13.1 (3.1–218)	6.4 (1.2–43.6)**	9.5 (1.2–113)**^††^
Prostate volume, ml	24.8 (6.9–69.9)	26.0 (8.7–48.8)*	20.6 (7.7–52.6)**^††^
Gleason score, 6:7:8–10	15:67:41	116:113:1**	20:126:59^††^
T stage, T1:T2:T3–4	47:37:39	131:98:1**	104:64:37**^††^
NCCN risk classification, low:intermediate:high	12:53:58	106:120:4**	4:113:88*^††^
Qmax, ml/s	9.9 (2.9–40.2)	12.3 (4.1–45.1)**	11.9 (3.8–35.6)**
PVR, ml	16.1 (0–147)	6.8 (0–216)**	7.8 (0–271)**
ADT			
No:Neo:Neo + adjuvant	35:16:71	140:86:4**	65:85:54**^††^

*P < 0.016; **P < 0.001 vs IMRT; ^†^ <0.016; ^††^*P* < 0.001 vs BT.

IMRT = intensity-modulated radiation therapy, BT = brachytherapy, 3D-CRT = 3D conformal radiation therapy, PSA = prostate-specific antigen, NCCN = National Comprehensive Cancer Network, Qmax = maximum flow rate, PVR = post-void residual, ADT = androgen-deprivation therapy, Neo = neoadjuvant therapy.

### Urinary domain

Chronological changes in urinary domain scores are shown in Table 2. The percentage of patients who lowered the score by the MID or more from baseline in the urinary domain at 3 months after BT, BT + 3D-CRT, and IMRT was 69%, 84% and 25%, respectively (Table 3). Multivariate analysis showed that, using the IMRT group as the reference, a greater number of patients in the BT [odds ratio (OR) = 6.28; 95% confidence interval (CI) = 2.94–13.4] and the BT + 3D-CRT (OR = 12.7; 95% CI = 6.02–26.9) groups showed a MID from baseline in the urinary domain at 3 months after treatment (Table 4). The percentage of patients who lowered the score by the MID or more from baseline at 24 months after BT, BT + 3D-CRT, and IMRT was 43%, 54% and 28%, respectively (Table 3). Multivariate analysis showed that, using the IMRT group as the reference, a greater number of patients of the BT group (OR = 2.22; 95% CI = 1.10–4.53) and the BT + 3D-CRT group (OR = 3.29; 95% CI = 1.67–6.45) showed a MID from baseline in the urinary domain at 24 months post-treatment (Table 4).

**Table 2.** Time-course of changes in EPIC questionnaire scores after intensity-modulated radiation therapy (IMRT), brachytherapy (BT), and BT + 3D conformal radiation therapy (3D-CRT) (BT+3D-CRT)

Domains	Baseline scores	Follow-up (months)				
		Scores (SD)				
		1	3	6	12	24
*n* =						
IMRT	123	123	123	121	121	121
BT	230	230	230	229	227	221
BT + 3D-CRT	205	205	204	203	205	201
Urinary summary						
Overall	95.3 (6.6)	85.3 (13.4)	85.4 (13.8)	89.7 (10.1)	92.4 (10.2)	89.7 (12.3)
IMRT	94.1 (7.4)	89.3 (10.5)	94.2 (7.4)	94.5 (6.5)	94.2 (8.1)	92.8 (10.0)
BT	95.9 (5.5)	83.2 (13.6)	85.5 (13.0)	88.4 (11.5)	92.5 (10.1)	90.4 (11.5)
BT + 3D-CRT	95.5 (7.2)	85.2 (14.2)	80.1 (14.7)	88.3 (11.8)	91.3 (11.2)	87.0 (13.8)
(1) Urinary function						
Overall	96.0 (8.4)	85.1 (16.3)	86.2 (15.7)	89.9 (13.4)	92.4 (12.6)	88.6 (16.0)
IMRT	94.0 (10.0)	89.2 (13.3)	94.6 (9.6)	94.4 (9.3)	94.5 (10.3)	92.2 (13.8)
BT	96.5 (7.4)	82.6 (16.6)	86.7 (15.3)	88.7 (13.5)	92.3 (12.1)	89.5 (14.7)
BT + 3D-CRT	96.5 (8.2)	85.3 (17.1)	80.8 (16.7)	88.6 (14.7)	90.9 (14.0)	85.5 (18.1)
(2) Urinary bother						
Overall	95.1 (7.2)	84.3 (14.6)	82.2 (15.5)	88.1 (12.2)	92.0 (10.9)	90.3 (11.9)
IMRT	94.1 (8.1)	89.4 (13.3)	93.9 (9.6)	94.6 (6.8)	94.0 (8.0)	93.2 (8.8)
BT	95.4 (6.6)	83.6 (14.3)	84.6 (14.3)	88.1 (12.4)	92.4 (11.8)	90.9 (11.3)
BT + 3D-CRT	94.7 (7.9)	85.1 (14.7)	79.5 (16.3)	88.0 (11.9)	91.7 (11.1)	88.0 (13.5)
(3) Urinary irritation						
Overall	97.3 (5.1)	84.4 (15.4)	84.8 (14.6)	91.0 (11.1)	93.5 (10.6)	92.1 (11.7)
IMRT	95.8 (7.5)	91.7 (9.8)	96.3 (5.7)	96.6 (6.1)	95.8 (7.7)	95.8 (7.3)
BT	97.5 (4.8)	82.7 (15.8)	87.1 (13.5)	87.1 (13.5)	94.0 (9.9)	92.5 (11.2)
BT + 3D-CRT	97.0 (5.4)	86.4 (14.8)	82.2 (15.3)	91.4 (10.2)	93.1 (11.2)	89.4 (13.2)
(4) Urinary incontinence						
Overall	96.0 (9.9)	89.0 (16.4)	85.7 (18.5)	88.8 (17.1)	92.9 (14.7)	89.2 (17.7)
IMRT	94.5 (10.9)	90.0 (15.8)	94.1 (12.1)	94.4 (10.9)	94.6 (12.0)	90.1 (16.1)
BT	96.1 (8.7)	89.0 (15.6)	88.2 (17.6)	89.1 (16.2)	93.6 (13.7)	90.3 (16.3)
BT + 3D-CRT	95.8 (11.0)	89.0 (17.3)	83.1 (19.1)	88.5 (18.1)	92.1 (15.6)	87.0 (19.8)
Bowel summary						
Overall	95.3 (6.2)	92.4 (8.4)	90.9 (9.8)	92.8 (8.0)	93.0 (8.1)	92.6 (9.2)
IMRT	95.1 (7.1)	91.0 (9.2)	93.4 (12.1)	95.0 (5.8)	91.6 (9.3)	90.4 (13.0)
BT	95.5 (5.6)	92.8 (8.3)	93.0 (8.1)	93.1 (8.0)	94.5 (6.3)	94.9 (6.6)
BT + 3D-CRT	95.1 (6.3)	92.8 (8.0)	87.1 (11.7)	91.4 (8.8)	92.2 (8.8)	91.6 (8.9)
(1) Bowel function						
Overall	93.9 (8.7)	88.8 (11.4)	86.9 (12.4)	89.5 (10.9)	89.7 (11.0)	89.4 (11.7)
IMRT	97.4 (7.3)	87.0 (12.0)	90.0 (9.7)	92.4 (8.8)	87.9 (12.3)	87.7 (14.6)
BT	93.3 (8.3)	89.3 (11.1)	89.5 (11.1)	89.8 (10.6)	91.4 (9.3)	92.0 (9.3)
BT + 3D-CRT	92.5 (9.3)	89.2 (11.3)	82.2 (13.6)	87.5 (11.8)	88.7 (11.7)	87.7 (11.8)
(2) Bowel bother						
Overall	97.7 (5.1)	96.0 (7.1)	94.9 (8.9)	96.2 (6.7)	96.4 (6.6)	95.8 (8.6)
IMRT	97.4 (7.3)	94.5 (7.9)	96.8 (5.3)	97.5 (3.8)	95.3 (7.8)	93.1 (12.7)
BT	97.8 (4.3)	96.2 (7.2)	96.3 (7.0)	96.5 (6.6)	97.6 (4.6)	97.6 (5.2)
BT + 3D-CRT	97.9 (4.2)	96.4 (6.3)	92.0 (11.6)	95.3 (8.4)	95.3 (8.7)	95.4 (8.1)
Sexual summary						
Overall	38.5 (13.0)	35.5 (10.4)	35.8 (11.4)	36.1 (10.9)	36.4 (11.5)	36.2 (12.1)
IMRT	38.2 (11.5)	33.9 (9.6)	33.8 (9.6)	33.5 (8.7)	33.5 (8.4)	32.5 (10.3)
BT	40.8 (14.2)	36.9 (10.4)	37.8 (12.5)	38.0 (12.0)	38.6 (12.8)	38.6 (13.5)
BT + 3D-CRT	36.2 (12.1)	35.0 (10.6)	34.7 (10.8)	35.5 (10.6)	35.6 (11.1)	35.7 (10.9)
Hormone summary						
Overall	92.9 (8.6)	92.8 (8.7)	93.3 (8.1)	94.2 (7.8)	94.2 (7.8)	94.4 (8.0)
IMRT	95.1 (7.1)	92.1 (8.4)	92.8 (7.8)	93.1 (8.2)	93.3 (7.0)	92.0 (9.3)
BT	93.1 (8.3)	94.5 (7.5)	94.7 (7.1)	95.2 (6.6)	95.9 (5.5)	96.4 (5.0)
BT + 3D-CRT	91.2 (9.3)	91.4 (9.6)	91.9 (9.1)	93.7 (8.7)	92.9 (9.4)	93.4 (9.3)

EPIC = Expanded Prostate Cancer Index Composite, IMRT = intensity-modulated radiation therapy, BT = brachytherapy, 3D-CRT = 3D conformal radiation therapy, IPSS = International Prostate Symptom Score, QOL = quality of life, SD = standard deviation.

**Table 3.** Percentage of patients who showed a decrease (minimally important difference) in quality-of-life scores from own baseline scores

Domains	Follow-up (months)				
	% (n)				
	1	3	6	12	24
Urinary summary					
IMRT	36 (44/123)	25 (31/123)	24 (29/121)	26 (31/121)	28 (34/121)
BT	73 (165/227)	69 (156/227)	60 (136/227)	35 (79/227)	43 (95/221)
BT + 3D-CRT	64 (131/205)	84 (173/205)	58 (118/205)	40 (83/205)	54 (114/201)
Bowel summary					
IMRT	52 (64/123)	41 (50/123)	33 (40/121)	45 (54/121)	43 (52/121)
BT	40 (91/227)	37 (84/227)	37 (85/227)	31 (70/227)	29 (64/221)
BT + 3D-CRT	39 (80/205)	68 (139/205)	46 (95/205)	41 (84/205)	46 (92/201)
Sexual summary					
IMRT	31 (22/123)	23 (28/123)	25 (30/121)	21 (25/121)	28 (34/121)
BT	27 (62/227)	23 (52/227)	26 (58/227)	26 (58/227)	28 (62/221)
BT + 3D-CRT	15 (30/205)	17(34/205)	15 (30/205)	18 (36/205)	16 (33/201)
Hormone summary					
IMRT	40 (49/123)	34 (42/123)	32 (39/121)	36 (44/121)	32 (39/121)
BT	21(40/227)	22 (49/227)	18 (40/227)	15 (35/227)	14 (31/221)
BT + 3D-CRT	25 (51/205)	24 (49/205)	19 (39/205)	22 (46/205)	23 (47/201)

EPIC = Expanded Prostate Cancer Index Composite, IMRT = intensity-modulated radiation therapy, IPSS = International Prostate Symptom Score, QOL = quality of life, BT = brachytherapy, 3D-CRT = 3D conformal radiation therapy, SD = standard deviation.

**Table 4.** Uni- and multivariate analysis of factors to predict a decrease (minimally important difference) in urinary domain scores from own baseline score at 3 and 24 months

Variables	3 months after radiation therapy			24 months after radiation therapy		
	Analysis					
	Univariate	Multivariate		Univariate	Multivariate	
	*P*	OR (95% CI)	*P*	*P*	OR (95% CI)	*P*
Age	0.31	0.98 (0.94–1.02)	0.22	0.15	0.97 (0.94–1.01)	0.077
Prostate volume	0.04	1.02 (0.99–1.05)	0.09	0.43	1.10 (0.99–1.03)	0.44
NCCN risk classification						
Low	Ref	Ref		Ref	Ref	
Intermediate	0.48	1.31 (0.74–2.33)	0.36	0.08	1.60 (0.93–2.76)	0.09
High	0.88	0.93 (0.36–2.42)	0.88	0.22	1.11 (0.49–2.53)	0.81
Qmax	0.78	0.98 (0.94–1.01)	0.23	0.85	0.98 (0.95–1.02)	0.3
PVR	0.02	1.00 (0.99–1.01)	0.56	0.18	1.00 (0.99–1.01)	0.4
ADT						
No	Ref	Ref		Ref	Ref	
Neo	<0.01	2.40 (1.38–4.2)	0.002	0.04	1.27 (0.80–2.01)	0.32
Neo+adjuvant	0.51	2.59 (1.04–6.48)	0.041	0.49	1.75 (0.82–3.72)	0.15
Radiation therapy						
IMRT	Ref	Ref		Ref	Ref	
BT	<0.01	6.28 (2.94–13.4)	<0.001	<0.01	2.22 (1.10–4.53)	0.027
BT + 3D-CRT	<0.01	12.7 (6.02–26.9)	<0.001	<0.01	3.29 (1.67–6.45)	0.001
Baseline domain	0.04	1.04 (1.01–1.08)	0.033	0.73	0.97 (0.94–1.01)	0.099

IMRT = intensity-modulated radiation therapy, BT = brachytherapy, 3D-CRT = 3D conformal radiation therapy, Qmax = maximum flow rate, PVR = post-void residual, ADT = androgen-deprivation therapy, Neo = neoadjuvant therapy, NCCN = National Comprehensive Cancer Network, CI = confidence interval, OR = odds ratio.

### Bowel domain

Chronological changes in bowel domain scores are shown in Table 2. The percentage of patients who lowered the score by a MID or more from baseline in the bowel domain at 3 months after BT, BT + 3D-CRT, and IMRT was 37%, 68% and 41%, respectively (Table 3). In multivariate analysis, using the BT group as the reference, a greater number of patients in the BT + 3D-CRT group (OR = 4.20; 95% CI = 2.53–6.98) showed a MID from baseline at 3 months after treatment. whereas, there was no significant difference between the IMRT group and the BT group (OR = 1.16; 95% CI = 0.65–2.10) (Table 5). The percentage of patients who lowered the score by a MID or more from baseline in the bowel domain at 24 months after BT, BT + 3D-CRT, and IMRT was 29%, 49% and 43%, respectively (Table 3). Multivariate analysis showed that, using the BT group as a reference, a greater number of patients in the IMRT (OR = 1.98; 95% CI = 1.07–3.67) and the BT + 3D-CRT (OR = 2.63; 95% CI = 1.57–4.42) groups showed a MID from baseline in the bowel domain at 24 months post-treatment (Table 5).

**Table 5.** Uni- and multivariate analysis of factors to predict occurrence of a decrease (minimally important difference) in bowel domain from own baseline score at 3 and 24 months post-treatment

Variables	3 months after radiation therapy			24 months after radiation therapy		
	Analysis					
	Univariate	Multivariate		Univariate	Multivariate	
	*P*	OR (95% CI)	*P*	*P*	OR (95% CI)	*P*
Age	0.88	0.99 (0.97–1.03)	0.94	0.01	1.05 (1.01–1.08)	0.006
Prostate volume	0.17	1.01 (0.99–1.03)	0.49	0.42	1.02 (0.99–1.04)	0.08
NCCN risk classification						
Low	Ref	Ref		Ref	Ref	
Intermediate	0.04	0.99 (0.97–1.03)	0.99	0.68	0.78 (0.45–1.35)	0.38
High	<0.01	1.21 (0.56–2.63)	0.62	0.07	0.86 (0.39–1.86)	0.69
ADT						
No	Ref	Ref		Ref	Ref	
Neo	0.66	0.76 (0.49–1.18)	0.22	0.04	1.10 (0.75–1.63)	0.63
Neo+adjuvant	0.35	0.69 (0.35–1.38)	0.29	0.49	1.21 (0.77–1.92)	0.41
Radiation therapy						
BT	Ref	Ref		Ref	Ref	
IMRT	0.38	1.16 (0.65–2.10)	0.61	<0.01	1.98 (1.07–3.67)	0.029
BT + 3D-CRT	<0.01	4.20 (2.53–6.98)	<0.001	<0.01	2.63 (1.57–4.42)	<0.001
Baseline domain	<0.01	1.08 (1.04–1.12)	<0.001	<0.01	1.08 (1.04–1.12)	<0.001

IMRT = intensity-modulated radiation therapy, BT = brachytherapy, 3D-CRT = 3D conformal radiation therapy, NCCN = National Comprehensive Cancer Network, Qmax = maximum flow rate, PVR = post-void residual, ADT = androgen-deprivation therapy, Neo = neoadjuvant therapy, CI = confidence interval, OR = odds ratio.

### Sexual domain

Chronological changes in the sexual domain score are shown in Table 2. The percentage of patients with a MID from baseline in the sexual domain at 3 months after BT, BT + 3D-CRT, and IMRT were 23%, 17% and 23%, respectively (Table 3). Multivariate analysis showed that at 3 months post-treatment, treatment modality was not a significant factor for MID from baseline in sexual QOL (Table 6). The percentage of patients who lowered the score by a MID or more from baseline in the sexual domain at 24 months after BT, BT + 3D-CRT, and IMRT was 28%, 16% and 28%, respectively (Table 3). Multivariate analysis showed that, using the BT +3D-CRT groups as a reference, a greater number of patients in the IMRT group (OR = 3.41; 95% CI = 1.71–6.82) showed a MID difference from baseline at 24 months after treatment.(Table 6).

**Table 6.** Uni- and multivariate analysis of factors to predict occurrence of a decrease (minimally important difference) in sexual domain scores from own baseline scores at 3 and 24 months post-treatment

Variables	3 months after radiation therapy			24 months after radiation therapy		
	Analysis					
	Univariate	Multivariate		Univariate	Multivariate	
	*P*	OR (95% CI)	*P*	*P*	OR (95% CI)	*P*
Age	<0.01	1.00 (0.96–1.05)	0.93	0.01	1.06 (1.01–1.11)	0.025
Prostate volume	<0.01	1.03 (1.01–1.06)	0.009	0.42	1.03 (1.002–1.05)	0.037
NCCN risk classification						
Low	Ref	Ref		Ref	Ref	
Intermediate	0.04	1.74 (0.88–3.46)	0.11	0.68	0.92 (0.47–1.78)	0.8
High	<0.01	1.34 (0.45–3.99)	0.6	0.07	0.90 (0.31–2.63)	0.84
ADT						
No	Ref	Ref		Ref	Ref	
Neo	0.49	1.23 (0.61–2.46)	0.56	0.04	0.55 (0.28–1.08)	0.08
Neo+adjuvant	0.17	2.49 (0.94–6.59)	0.07	0.49	1.30 (0.49–3.42)	0.6
Radiation therapy						
BT + 3D-CRT	Ref	Ref		Ref	Ref	
IMRT	0.15	0.92 (0.43–1.98)	0.84	<0.01	3.41 (1.71–6.82)	<0.001
BT	0.12	1.15 (0.59–2.24)	0.69	<0.01	1.26 (0.63–2.49)	0.51
Baseline domain	<0.01	1.10 (1.07–1.12)	<0.001	<0.01	1.11 (1.08–1.14)	<0.001

IMRT = intensity-modulated radiation therapy, BT = brachytherapy, 3D-CRT = 3D conformal radiation therapy, NCCN = National Comprehensive Cancer Network, Qmax = maximum flow rate, PVR = post-void residual, ADT = androgen-deprivation therapy, Neo = neoadjuvant therapy, CI = confidence interval, OR = odds ratio.

### Hormone domain

Chronological changes in hormone domain scores are shown in Table 2. The percentage of patients who lowered the score by a MID or more from baseline in the hormonal domain at 3 months after BT, BT + 3D-CRT, and IMRT was 22%, 24% and 34%, respectively (Table 3). The percentage of patients who lowered the score by a MID or more from baseline in the sexual domain at 24 months after BT, BT + 3D-CRT, and IMRT was 14%, 23% and 32%, respectively (Table 3). Multivariate analysis showed that at 3 months and at 24 months post-treatment, treatment modality was not a significant factor for hormone domain QOL changes (Table 7).

**Table 7.** Uni- and multivariate analysis of factors to predict occurrence of a decrease (minimally important difference) in hormone domain scores from own baseline scores at 3 and 24 months post-treatment

Variables	3 months after radiation therapy			24 months after radiation therapy		
	Analysis					
	Univariate	Multivariate		Univariate	Multivariate	
	*P*	OR (95% CI)	*P*	*P*	OR (95% CI)	*P*
Age	0.4	0.99 (0.96–1.03)	0.78	0.03	1.02 (0.98–1.06)	0.3
Prostate volume	0.13	1.02 (1.00–1.04)	0.049	0.85	1.01 (0.98–1.03)	0.74
NCCN risk classification						
Low	Ref	Ref		Ref	Ref	
Intermediate	0.29	1.25 (0.68–2.29)	0.48	0.02	1.69 (0.81–3.54)	0.16
High	0.04	1.17 (0.49–2.79)	0.73	<0.01	3.07 (1.14–8.28)	0.027
ADT						
No	Ref	Ref		Ref	Ref	
Neo	0.19	1.95 (1.17–3.31)	0.011	0.04	0.58 (0.31–1.07)	0.08
Neo+adjuvant	<0.01	2.86 (1.35–6.05)	0.006	0.01	0.89 (0.40–1.98)	0.78
Radiation therapy						
BT	Ref	Ref		Ref	Ref	
IMRT	0.04	0.94 (0.48–1.86)	0.87	<0.01	1.34 (0.65–2.76)	0.43
BT + 3D-CRT	0.5	1.07 (0.61–1.89)	0.82	<0.01	1.53 (0.82–2.85)	0.18
Baseline domain	0.04	1.07 (1.03–1.10)	<0.001	<0.01	1.08 (1.04–1.13)	<0.001

IMRT = intensity-modulated radiation therapy, BT = brachytherapy, 3D-CRT = 3D conformal radiation therapy, NCCN = National Comprehensive Cancer Network, Qmax = maximum flow rate, PVR = post-void residual, ADT = androgen-deprivation therapy, Neo = neoadjuvant therapy, CI = confidence interval, OR = odds ratio.

## DISCUSSION

To the best of our knowledge, ours is the first study to comparatively evaluate the effect of BT, BT + 3D-CRT, and IMRT on patient QOL. Evans *et al.* found that BT caused worse urinary irritation at 2 years (*P* < 0.0001) than did IMRT [3]. The ASCENDE-RT trial showed that a low-dose-rate prostate brachytherapy boost lowered urinary function to a greater extent than did a dose-escalated external beam boost (−3.6 vs −0.5; *P* = 0.04) [12]. In agreement with the above, in the present study, at 3 and 24 months after treatment, BT + 3D-CRT was found to lower urinary QOL scores most severely, followed by BT.

In the bowel domain, compared with the BT group, significantly lower QOL scores were seen in the BT + 3D-CRT (3 and 24 months post-treatment), and IMRT groups (24 months post-treatment), on multivariate analysis. Evans *et al.* reported there was no significant difference in bowel-related QOL between IMRT and BT [3], but Ferrer *et al.*, in agreement with our results, reported that EBRT led to significantly worse bowel summary scores than did BT (*P* < 0.001) [13]. The discrepancy in results may be accounted for by the relatively lower QOL scores reported by Evans *et al.* after BT compared with in the other studies [13, 14], including the present study. The ASCENDE-RT trial showed that there was no significant difference in bowel-related QOL between low-dose-rate prostate brachytherapy boost and a dose-escalated external beam boost [12]. In the present study, the difference between IMRT and BT + 3D-CRT was not evaluated statistically in the bowel domain. However, in agreement with the ASCENDE-RT trial results, the percentages of the patients with a MID in the IMRT and BT + 3D-CRT groups were similar between IMRT (43%) and BT+3D-CRT (46%) at 24 months. Considering the above evidence and the present results, out of BT, BT + 3D-CRT, and IMRT, BT + 3D-CRT may lower bowel QOL most severely, followed by IMRT.

In the sexual domain, IMRT lowered QOL more severely at 24 months post-treatment, compared with BT + 3D-CRT. Although Evans *et al.* reported no significant differences between the IMRT and BT groups [3], Spratt *et al.* found that sexual QOL scores were similar between the IMRT and BT + IMRT groups (57.8% vs 55.0%; *P* = 0.67) [15]. The ASCENDE-RT trial reported lower sexual QOL scores due to a low-dose-rate prostate brachytherapy boost (−22.1 points) than due to a dose-escalated external beam boost (−15.3 points) at 24 months [12]. Although the discrepancy between the above and the present results is difficult to explain, there are a number of possible reasons. The baseline score for the sexual domain in the present study was lower compared with in the other studies, and the patients in the present study were older than those in the other studies [3, 12, 15]. Age and the sexual QOL at baseline are important factors affecting sexual QOL after radiation therapy [4], like the present results (Table 6). Therefore, the differences between the populations may have caused the discrepancy.

In the hormone domain, radiation treatment did not predict a lower EPIC score in the present study at 3 months or 24 months post-treatment. Evans *et al.* reported that there were no significant differences in hormone-related QOL between seed and IMRT [3], although they did not include androgen-deprivation therapy (ADT) as a factor in the multivariate analysis, which may have affected the results.

BT + 3D-CRT is known to lead to a better prognosis in intermediate- and high-risk prostate cancer [5, 6]; however, QOL in the urinary and bowel domains was reduced by BT + 3D-CRT, especially at 3 months post-treatment. Therefore, knowledge of changes in QOL outcomes due to IMRT, BT, and BT + 3D-CRT may guide treatment recommendations and enable patients to make better-informed decisions. Furthermore, patients who undergo BT + 3D-CRT should be offered some treatments for lowered QOL in the 3 months following treatment, to address lower urinary tract symptoms (LUTSs) and bowel function. However, there are few studies on treatments for LUTS and bowel function problems caused by radiation therapy. Calcium-channel blockers and statins for acute rectal toxicity [16], and an alpha-1 blocker or anticholinergic drug for LUTS should be evaluated for improving QOL in future studies [10, 17].

The present study had some limitations. The first limitation was the lack of randomization for type of treatment, which may have led to the possibility that unmeasured selection factors may have influenced the outcomes. Second, the follow-up period was short, considering that the 10-year overall survival rate of patients treated with radiation therapy is relatively high (>70%) [18, 19]. The third limitation was the use of 3D-CRT as a boost after BT. Forsythe *et al.* reported that BT+3D-CRT lowered urinary QOL more severely than did BT + IMRT (*P* < 0.001) [20]. In future studies, a longer follow-up period and inclusion of a BT + IMRT group is indicated.

## CONCLUSIONS

Out of BT, BT + 3D-CRT, and IMRT, BT + 3D-CRT lowered urinary and bowel QOL most severely. BT lowered urinary QOL more severely compared with IMRT, and IMRT lowered bowel QOL more severely compared with BT. Knowledge of changes in QOL outcomes associated with IMRT, BT, and BT + 3D-CRT could influence treatment recommendations and enable patients to make better-informed decisions.
